# Chedoke-McMaster attitudes towards children with handicaps scale for traditional sporting games (CATCH-TSG): initial validation in 7 different languages in adult and young populations

**DOI:** 10.3389/fpsyg.2023.1254580

**Published:** 2023-09-25

**Authors:** Jaume March-Llanes, Lluis Mas-Ruiz, Jorge Moya-Higueras, Josep Rius-Torrentó, Veronica Estrada-Plana, Judit Bañeres, Pere Lavega-Burgués, The Opportunity Team, R Camacho, C Duran, M Esperanza, C Fernández-Amat, I Gracia, F Lagardera, C Mallén, V Muñoz-Arroyave, E Ormo, P Pla, Q Prat, R Rodriguez-Arregi, P Ruiz, U Sáez de Ocáriz, B Soria, AR Jaqueira, P Coelho de Araújo, A Romao, MM Duarte, Z Ben Chaâbanne, E Bouzid, PG Avigo, F Berti, L Carcereri, F Berti, G Torresani, T Đerić Nikolić, M Pacenti, T Perković, B Prabucki, K Waluch, Z Kuznetsova

**Affiliations:** ^1^Department of Psychology, University of Lleida, Lleida, Spain; ^2^Institut de Recerca de Desenvolupament Social i Territorial (INDEST), University of Lleida, Lleida, Spain; ^3^University of Lleida, Computer Engineering and Digital Design, Lleida, Spain; ^4^Centre for Biomedical Research Network on Mental Health (CIBERSAM), Instituto de Salud Carlos III, Barcelona, Spain; ^5^Department of Economy and Business, University of Lleida, Lleida, Spain; ^6^National Institute of Physical Education of Catalonia (INEFC), Motor Action Research Group (GIAM), INDEST, University of Lleida, Lleida, Spain

**Keywords:** stereotype, attitude, disability, social inclusion, cultural diversity

## Abstract

**Introduction:**

Measuring attitudes towards disability is meant to assess which interventions are most likely to create changes in population attitudes. Physical activities, such as Traditional Sports Games, are an excellent methodology to fight against the stigma of disabled people. Thus, the main aim of this study was to validate the Chedoke-McMaster Attitudes towards Children with Handicaps Scale (CATCH) adapted to a physical activity environment.

**Methods:**

Additionally, we implemented this process in a combined way, 7 languages and 2 versions (adult and youth) at the same time.

**Results:**

The results showed that the CATCH-TSG scale provides the scientific community with a valid and reliable tool for professionals who want to assess the change in attitudes towards people with disabilities after receiving a psychoeducational intervention that includes physical activity (TSG).

**Discussion:**

Researchers will compare respective results from different countries and ages using different versions of the scale, jointly validated.

## 1. Introduction

### 1.1. Persons with disabilities make up the world’s largest and most disadvantaged minority

People with disabilities remain amongst the most marginalized in every society. Over 650 million persons around the world live with disabilities. Persons with disabilities make up the world’s largest and most disadvantaged minority, in most parts of the world, there are deep and persistent negative stereotypes and prejudices against persons with certain conditions and differences. These attitudes determine who is considered to be a person with a disability and perpetuate the negative image of persons with disabilities (UN, 2007, p.3).

Nowadays, discrimination and negative stereotypes pose significant obstacles to the full participation and equity of individuals with intellectual disabilities in society. The transformation of stereotypes related to individuals with intellectual disabilities requires global, national, and local actions. International guidelines provide guidance and frameworks for transforming stereotypes targeting individuals with intellectual disabilities. The UN’s Agenda 2030, with its 17 Sustainable Development Goals (SDGs), prioritises promoting inclusion, equal opportunities, and eliminating discrimination against individuals with intellectual disabilities. SDGs 4, 8, and 10 prioritise ensuring inclusive, equitable, and quality education, free from stereotypes. The International Convention on the Rights of Persons with Disabilities (CRPD), adopted by the UN General Assembly in 2006, sets out the fundamental human rights of people with disability and had the purpose to promote, protect and ensure the full and equal enjoyment of all human rights and fundamental freedoms by all persons with disabilities, and to promote respect for their inherent dignity. CPRD emphasises the need to change attitudes and negative stereotypes towards individuals with disabilities, including intellectual disabilities.

### 1.2. Stereotypes referred to people with intellectual disabilities

Stereotypes and attitudes are synonyms when they refer to a specific social group. Eagly and Mladinic (1989) defined attitudes as the tendency to evaluate an entity with a certain degree of favour or disfavour. These same authors, joint with more recent ones (Bohner and Dickel, 2011; Crano and Gardikiotis, 2015), assume that attitudes should be understood as stereotypes when the entity is a social group. Thus, a stereotype is a tendency to evaluate a social group with a certain degree of favour or disfavour.

According to the World Health Organisation, disability is an impairment in a person’s body, mental structure or function, significantly limits in his/her activities (WHO, 2001). These obstacles do not allow disabled people to participate in normal daily activities (WHO, 2001). And finally, the participation restrictions are usually linked to stereotypes that non-disabled people have about them (WHO, 2001). Hence, attitudes (stereotypes) towards disabled people are a core concept of disability.

Crano and Gardikiotis (2015) and Eagly and Mladinic (1989) propose that attitudes (or stereotypes when we speak about a social group) are composed of interaction of cognitive, emotional, and behavioural factors. The first author to propose this model was Triandis (1971). Cognitive variables are related to the beliefs regarding the social group. Emotional factors come from the emotional experiences that the person has with people from the social group. Besides, this factor is also related to our estimations regarding how people from the social group feel in affective situations. Finally, the behavioural component is linked with specific behaviours we do or we want to do with people from the social group and the expectancies we have about how people from the social group can behave. In empiric studies, the Triandis model best predicts of different outcomes (Valois et al., 1988).

### 1.3. Traditional games. A tool for transforming stereotypes in educational contexts

The approach to education promoted by the UN CRPD Convention (2007)is based on a growing body of evidence that shows that inclusive education not only. Provides the best educational environment, including for children with intellectual disabilities, but also helps to break down barriers and challenge stereotypes. This approach helps to create a society that readily accepts and embraces disability, instead of fearing it. When children with and without disabilities grow up together and learn, side by side, in the same school, they develop a greater understanding and respect for each other (UN, 2007, p.3).

This study is grounded in the theoretical principle that participation in appropriate Traditional Sports Games (TSGs) programmes can alter participants’ attitudes associated with negative stereotypes, which often encompass prejudices and erroneous beliefs about individuals with disabilities and the female gender.

Multiple empirical pieces of evidence demonstrate that attitudes are acquired and can be modified through intervention programmes (Triandis, 1971; Rosenbaum et al., 1986b; Eagly and Mladinic, 1989; McDougall et al., 2004; Bohner and Dickel, 2011; Cameron et al., 2011; McKay et al., 2015; Mirnezami et al., 2015; de Ocariz Granja and Lavega Burgues, 2015). Amongst the theories that explain the promotion of attitude transformation, this project draws upon contact theory (Allport, 1954; Pettigrew, 1998). According to Allport’s Intergroup Contact Hypothesis (Allport, 1954), the main way to change attitudes is to put in touch people from diverse groups. It is believed that contact with diverse individuals tends to induce attitude changes when presented within a context of institutional support that encompasses three conditions (McKay, 2018; McKay et al., 2021): (a) Equality of status (wherein the game rules are the same for all participants); (b) Pursuit of common goals (wherein the inherent logic of the game poses similar problems to be solved); (c) Meaningful personal interactions (wherein individuals who engage in the game tend to bring their complete organic, affective, relational, and cognitive experiences, Pic et al., 2019).

In addition to those above, attitudes stem from both a personal evaluative construct and a normative construct that emerge from behaviours occurring within social interaction contexts (Triandis, 1971; Schmidt and Rakoczy, 2019). Based on these scientific arguments, it seems reasonable to utilize TSGs to promote inclusive attitudes within an educational intervention programme. if adequately implemented, TSGs, as conduits for interpersonal relationship experiences founded on the democratic acceptance of rules and physical interaction between participants (often involving intense motor interaction with physical contact), can serve as excellent educational resources to transform potential attitudes that do not foster gender equality and social inclusion within the framework of intercultural physical education.

In the educational context, we start from the assumption that students’ attitudes—for example, their behavioural intentions—are influencing social participation (Crano and Gardikiotis, 2015; Marín-Suelves and Ramón-Llin, 2021). The practise of Traditional Games and Sports (TSG) in formal contexts could favour the transformation of participants’ attitudes regarding issues of core goals as social inclusion of disabled people.

### 1.4. Measuring attitudes towards people with disabilities

Attitudes are difficult to measure directly, so self-report scales are used as measures for the study of prejudice (Armstrong et al., 2017). From the 70s studies began to appear on the attitudes of subjects towards those with disabilities and began to raise awareness about the importance of producing changes in attitudes in the population (Santiago García et al., 2003). These are essential to be able to change people’s attitudes towards people with disabilities or mental disorders. Some of the scales highlighted in the literature that have been used by various authors, such as the *Reported and Intended Behaviour Scales* (RIBS; Evans-Lacko et al., 2011; Evans-Lacko et al., 2013), were not based on the Triandis model. Then, Rosenbaum et al. (1986a,b) created the Chedoke–McMaster Attitudes towards Children with Handicaps (CATCH) scale to fill this gap. However, they did not find a three factor structure, as it was predicted by the Triandis (1971) model. Later studies have found diverse results (De Boer et al., 2012; Bossaert and Petry, 2013; Armstrong et al., 2017). In fact, these more recent studies have tried to reduce the number of items used (the initial measure consisted of 36 statements). Hence, though it has been used, the standard CATCH measure has received mixed results regarding its validity and its reliability. Even though, there is a consensus regarding the usefulness of the CATCH to assess attitudes, as it has been used in research up to 16 years, being recommended as a reliable, valid and complete measure (Vignes et al., 2008). In fact it is one of the few scales that evaluate the effectiveness of programmes designed to promote positive attitudes towards their people with disabilities (Tavares, 2011). Thus, the CATCH seem to be one of the most complete instruments amongst those identified in the review made by Vignes et al. (2008) as they include all three attitude components and have appropriate psychometric properties.

### 1.5. Opportunity project. Networking for transforming stereotypes

Opportunity is a co-funded project by the Erasmus Programme of the European Commission (EACEA). Nine partners from 7 countries (Spain, France, Portugal, Italy, Croatia, Poland and Tunisia) as well as several associated partners from Russia and other continents have been sharing the efforts from January 2021. The project aims to promote of Traditional Games and Sports (TSG) as a tool for fostering social inclusion of people with intellectual disabilities within a variety of educational scenarios.

Opportunity project offers an opportunity for networking of organisations and professionals from different countries and continents interested in transforming stereotypes about people with disabilities through TSGs. This project provides a free APP with the CATCH-TSG questionnaire translated and adapted to other languages.

As far as we now, no previous attitudes towards disabled people’s test have been adapted to physical activities. Hence, the objective of the present study was to adapt some items of the CATCH scale to generate the CATCH-TSG (CATCH applied to Physical Activity by using Traditional Sporting Games) to fit the physical activity topic, proposing versions in 8 languages. In addition, we prepared two versions of the instrument, one more suitable to adults and another one better for adolescents. Then, we tested the validity and reliability of in all the languages. We hypothesised that the CATCH-TSG would be a valid and reliable measure to assess attitudes.

## 2. Methods

### 2.1. Sample

A total of 3,706 participants completed the CATCH-TSG. Participants were selected through a non-probabilistic process (incidental, snowball, and convenience). They answered first some sociodemographic items, membership of sports clubs, and aspects related to language, in order to detect responses made in an incorrect version of test.

The assessment was performed between March and September 2020. Adolescents and adults were measured in each of the 6 countries where the research was executed (in Tunisia we recruited 2 different samples, Arab and French). Table 1 shows basic demographic data. We had more responses in the adult version, with an overall percentage of women of 56% (male 43.4% and non-binary 0.6%).

**Table 1:** Demographic data.

Version	*n*	Mean age	SD age	Age maxim	Age minimum	%Female sex
Arab Adult	156	27.2	11.2	72.0	18.0	26.3%
Arab Youth	154	14.2	2.0	18.0	11.0	40.3%
French Adult	174	36.4	16.8	72.0	18.0	52.9%
French Youth	203	14.5	1.9	18.0	11.0	35.0%
Italian Adult	391	24.8	12.2	72.0	18.0	82.6%
Italian Youth	176	15.6	1.4	18.0	11.0	80.1%
Polish Adult	268	32.7	12.0	65.0	18.0	59.0%
Polish Youth	209	13.7	2.1	17.0	11.0	58.9%
Portuguese Adult	375	28.2	13.6	64.0	18.0	38.9%
Portuguese Youth	258	14.1	2.0	18.0	11.0	52.7%
Russian Adult	287	21.6	6.6	60.0	18.0	73.3%
Russian Young	163	16.2	1.0	18.0	14.0	60.1%
Spanish Adult	342	28.5	12.6	76.0	18.0	50.1%
Spanish Young	550	13.7	1.6	18.0	11.0	54.5%

### 2.2. Instrument

The CATCH scale was created by Rosenbaum et al. (1986b) as a self-administered scale for children ages 9 to 13 to measure attitudes toward their peers with disabilities. The CATCH consists of 36 items, 12 written negatively and the other half written positively, arranged in random order alternating positive and negative statements. Before administering the questionnaire, participants are told to interpret the word “disabled” as they understand it without presenting any specific stimulus for clarification. The rationale for this approach is that regardless of disability, children tend to have similar attitudes toward children with disabilities (Rosenbaum et al., 1986a). Finally, the answers are recorded using a 5-Likert scale (from strongly disagree to strongly agree; Armstrong et al., 2016).

### 2.3. Data retrieval

To collect the necessary information for this study, a survey website^1^ was developed. The website consisted of three main components: the front-end, developed using Angular^2^; the back-end, developed using NodeJS^3^ to handle communications and calculations; and the encrypted MongoDB^4^ database, which stored the data to ensure user privacy.

Accessibility was prioritised to accommodate a diverse range of users. To achieve this, a mobile-first design approach was adopted, and the application was translated into multiple languages. The use of neutral colours further enhanced usability for visually impaired individuals.

Additionally, an admin panel was implemented to enable supervisors to oversee and ensure the smooth functioning of the activity. The supervisors played a crucial role in providing feedback on the system, enabling iterative improvements over time.

Given the intermittent nature of survey demands, it was imperative for the system to support high loads efficiently. To address this, a container-based cloud hosting solution (Ruíz et al., 2022) was selected. This approach facilitated rapid scaling based on demand and subsequent resource downsizing, making the system both resource-friendly and environmentally sustainable.

To cater to a global user base, the system was translated into 10 languages and implemented real-time translation without incurring any downtime. Docker^5^ was chosen for building and deploying the system, offering seamless deployment capabilities.

Overall, the survey website’s design and infrastructure were carefully developed to ensure data collection, accessibility, supervisory oversight, scalability, and global availability.

### 2.4. Procedure

The CATCH adapted to a context of physical activity (CATCH-TSG) was created to assess the attitudes of children and adults towards disability in this setting. We generated the items to ask about physical activity situations using a language adapted to both versions (Youth and Adult). We produced the first adapted versions in English. Then, both forms were translated into the 7 study languages: Arab, French, Italian, Polish, Portuguese, Russian and Spanish. Native members of the research team conducted translations and back-translations.

The adaptation process necessitated modifications in approximately half of the items in the original version, precisely 36 items, with a specific focus on 8 out of the 19 items featured in the final iteration. These alterations were meticulously crafted to imbue each element with a contextual essence firmly grounded in the domain of Traditional Sports Games. Moreover, gender-specific references, thoughtfully calibrated to resonate with adult and young audiences, were deployed, incorporating each respective language’s most contemporary linguistic nuances. Our commitment to employing the most precise and inclusive language extended to our discussions surrounding disability across all languages involved in this endeavour.

Within the framework of the Opportunity study, each partner assumed the role of an autonomous panel responsible for their designated language, thereby affirming the validation of the translation and adaptation process through consensus. In each language, a team of two translators was engaged in the translation process from English to the respective local language. Subsequently, both translations were juxtaposed, and in the event of disparities, the two translators collaborated to forge a consensus-based rendition. This reconciled version was then entrusted to another pair of translators for the reverse translation into English, whereupon it was scrutinised against the original version. In cases where discrepancies persisted, all four translators collectively contributed to the ultimate formulation of the local version. Notably, Tunisian partner assumed responsibility for the adaptation efforts pertaining to both the French and Arabic languages, resulting in the exclusive utilization of these languages within the borders of Tunisia.

The meticulous design of the questionnaire administration procedure was underpinned by a commitment to flexibility and inclusivity across all participating countries. Within the expansive ambit of the Opportunity Project, the questionnaire was adeptly administered through a dedicated application (APP), guaranteeing accessibility and user-friendliness for all respondents. Regardless of their geographic location, individuals utilizing the application to partake in the questionnaire exercise were afforded the liberty to select the language that resonated most harmoniously with their sensibilities.

This innovative approach afforded respondents a spectrum of languages to choose from, thereby ensuring that the questionnaire was attuned to their linguistic predilections. Consequently, participants from Tunisia and other nations were empowered with the agency to complete the questionnaire in their language of choice, a pivotal stride towards fostering inclusivity and accommodating the rich tapestry of linguistic diversity woven by participants.

### 2.5. Statistical analyses

The statistical analysis corresponds to a psychometric study focused on reliability and construct validity combining Exploratory a Confirmatory Factor Analysis. The validation process involved several phases, including creating a version of CATCH adapted to a particular scenario, with 7 different languages and two distinct versions (youth, and adults). This combined procedure was explained below.

We first applied Exploratory Factor Analysis (EFA) with Principal Components Analysis, Maximum Likelihood extraction method, based on parallel analysis with Oblimin Rotations, in the more suitable sample. As the CATCH questionnaire (Rosenbaum et al., 1986a) was created for children and adolescent populations, we began the validation of CATCH-TSG with the youth version and with the larger sample, precisely the Spanish youth sample.

Once we established the factorial solution in the initial sample, we carried out Confirmatory Factor Analyses (CFA) to test whether the initial solution was replicated in the other 6 samples. We made different attempts to maximize the Fit indexes and reliability coefficients.

The model, which was derived through EFA, underwent a re-specification process aimed at refining its structure. In this endeavour, we incorporated an additional item into Factor 1, and augmented Factor 3 with the inclusion of two more items, with the overarching objective of attaining a commendable reliability coefficient of 0.6. Furthermore, we introduced residual covariances, extending up to four instances, in order to bolster the goodness-of-fit indices of the models subjected to CFA.

After youth version validation, we applied the same methodology (CFA) in all 7 adult samples.

When we finished the validation of 36-items versions, we initiated the validation of a reduced version for facilitating the usage of the CATCH-TSG in difficult contexts. So, first, we used EFA and CFA in the sample of Spanish adolescents. The goal was to obtain a 4-factor short version with 4 items per factor, with a reliability coefficient restriction that consisted in add items in the worst factors if needed.

Finally, we used CFA in all the other samples to replicate the shortened solution.

## 3. Results

### 3.1. Young population full model

With the initial EFA of 36 items we obtained a solution with 4 factors which explain the 41.85% total variance [Chi-square Bartlett’s (630) = 6345.88; *p* < 0.001. and KMO MSA Overall = 0.92]. Interpreting what the items shared in common in each factor we named the first one as “positive interpersonal attitudes” and the second one as “negative interpersonal attitudes,” both towards disabled people. Factor three was related to the cognitions that respondents had regarding disabled people, and because of this commonality we named this factor “social cognition.” Finally, the last factor was manly focused on the help that responders believe that disabled people need, so the fourth factor was named “behavioural help.” Table 2 presents the CFA goodness-of-fit statistics in the 7 languages. We also show the reliability coefficients of each factor in each language. As can be seen, the worst reliability in most samples corresponds to factor 3, and the best reliability corresponds to factor 1. The adjustments, based on the different criteria, range from a very good fit in Spanish to the worst results, obtained in Arab.

**Table 2:** Results obtained from the CFA of the 6 versions analyzed, in the complete questionnaire of 36 items for the youth version.

Language	$\chi^{2}$	D.f.	p	CFI	TLI	RMSEA	AIC	α-1	α-2	α-3	α-4
Spanish								0.89	0.73	0.54	0.67
Arab	643.88	310	<.001	0.732	0.697	0.084	11821.6	0.78	0.63	0.32	0.47
French^1^	584.25	304	<.001	0.848	0.824	0.067	15848.3	0.79	0.67	0.57	0.52
Italian	592.51	360	<.001	0.876	0.860	0.061	11238.4	0.87	0.82	0.51	0.47
Polish	557.90	310	<.001	0.881	0.865	0.062	13077.4	0.90	0.74	0.59	0.65
Portuguese	510.80	316	<.001	0.921	0.913	0.049	16143.9	0.91	0.81	0.64	0.67
Russian	505.49	313	<.001	0.762	0.733	0.061	12799.2	0.73	0.77	0.34	0.32

D.f. = Degrees of freedom; α-1: Cronbach’s alpha of factor 1; α-2: Cronbach’s alpha of factor 2; α-3: Cronbach’s alpha of factor 3; α-4: Cronbach’s alpha of factor 4. ^1^Tunisia.

### 3.2. Adult population full model

After the initial validation of the adolescent version, several CFAs were carried out in the 7 languages in adult samples. Table 3 shows the CFA goodness-of-fit statistics in all the 7 adult samples and also the reliability coefficients.

**Table 3:** Results obtained in the Spanish sample and in the 7 versions of the CFA in the complete questionnaire of 36 items for the adult population.

Language	$\chi^{2}$	D.f.	*p*	CFI	TLI	RMSEA	AIC	α-1	α-2	α-3	α-4
Spanish	579.55	310	<.001	0.891	0.876	0.050	19762.2	0.85	0.75	0.57	0.57
Arab	638.36	315	<.001	0.731	0.700	0.081	10628.2	0.82	0.72	0.51	0.59
French^1^	576.91	314	<.001	0.762	0.734	0.069	11934.5	0.78	0.74	0.33	0.59
Italian	589.62	314	<.001	0.912	0.901	0.047	21083.9	0.88	0.76	0.53	0.50
Polish	511.81	309	<.001	0.901	0.887	0.049	15970.7	0.87	0.75	0.54	0.56
Portuguese	589.72	312	<.001	0.914	0.903	0.049	22009.2	0.88	0.80	0.49	0.65
Russian	654.95	310	<.001	0.852	0.833	0.062	17999.2	0.84	0.77	0.61	0.52

D.f. = Degrees of freedom; α-1: Cronbach’s alpha of factor 1; α-2: Cronbach’s alpha of factor 2; α-3: Cronbach’s alpha of factor 3; α-4: Cronbach’s alpha of factor 4. ^1^Tunisia.

As can be seen, the pattern of results obtained is similar to the obtained results in Youth, the worst results refer to factor 3 and in the Arabic version, while the best results are obtained in the Spanish version.

### 3.3. Young population reduced model

We first established the constrain to maintain the initial 4 factor solution in the short version, and to use in a combined form, a reliability threshold criterium to generate this reduced version. The short version could not reduce Cronbach’s alpha values below 0.6 in any factor if in the full version, Cronbach alpha was higher than 0.6. Following these criteria, we obtained a 19 items solution with PCA in the young Spanish sample. This solution, explained the 49.67% of the variance [Chi-square Bartlett’s test (171) = 2473.86; *p* < 0.001. and KMO MSA Overall = 0.84]. Table 4 shows all the goodness-of-fit statistics and reliability in all the samples. As can be seen, the best reliability corresponds to factor 1 and the worst reliability, to factors 3 and 4 is almost 0.6. The adjustments, based on the different criteria, range from a very good adjustment in Portuguese and the worst results obtained in Arab.

**Table 4:** Results obtained in the Spanish sample and in the other six versions of the CFA in the shortened version of 19 items for the youth version.

Language	$\chi^{2}$	D.f.	p	CFI	TLI	RMSEA	AIC	α-1	α-2	α-3	α-4
Spanish								0.80	0.67	0.64	0.67
Arab	304.64	144	<.001	0.770	0.727	0.085	8295.5	0.64	0.39	0.55	0.47
French^1^	207.13	139	<.001	0.931	0.915	0.049	11431.7	0.63	0.61	0.73	0.52
Italian	208.33	140	<.001	0.928	0.912	0.053	7432.3	0.80	0.74	0.67	0.47
Polish	260.24	141	<.001	0.903	0.882	0.064	9190.4	0.80	0.73	0.72	0.65
Portuguese	228.67	145	<.001	0.933	0.921	0.047	11706.2	0.82	0.75	0.60	0.67
Russian	259.53	146	<.001	0.725	0.678	0.069	9110.1	0.47	0.67	0.44	0.32

D.f. = Degrees of freedom; α-1: Cronbach’s alpha of factor 1; α-2: Cronbach’s alpha of factor 2; α-3: Cronbach’s alpha of factor 3; α-4: Cronbach’s alpha of factor 4. ^1^Tunisia.

### 3.4. Adult population reduced model

Finally, as in the complete version, a CFA was carried out in the 7 samples to test the shortened version of the scale in the adult samples (See Table 5). The best reliability still corresponds to factor 1 and the worst reliability to factor 4. The adjustments, based on the different criteria, range from a very good adjustment in Portuguese and the worst results are again obtained in Arab.

**Table 5:** Results obtained in the Spanish sample and in the 7 versions of the CFA in the shortened version of 19 items for the adult population.

Language	$\chi^{2}$	D.f.	p	CFI	TLI	RMSEA	AIC	α-1	α-2	α-3	α-4
Spanish	260.37	136	<.001	0.918	0.897	0.052	14070.1	0.78	0.71	0.66	0.57
Arab	209.20	138	<.001	0.873	0.843	0.058	7563.7	0.67	0.60	0.60	0.59
French^1^	236.48	143	<.001	0.861	0.833	0.061	8482.53	0.67	0.67	0.54	0.59
Italian	273.82	144	<.001	0.925	0.911	0.048	15384.3	0.82	0.71	0.63	0.50
Polish	222.73	144	<.001	0.927	0.914	0.045	11531.9	0.77	0.70	0.65	0.56
Portuguese	272.313	142	<.001	0.929	0.915	0.049	15632.4	0.81	0.76	0.59	0.65
Russian	268.05	134	<.001	0.895	0.866	0.059	12832.4	0.73	0.68	0.71	0.52

D.f. = Degrees of freedom; α-1: Cronbach’s alpha of factor 1; α-2: Cronbach’s alpha of factor 2; α-3: Cronbach’s alpha of factor 3; α-4: Cronbach’s alpha of factor 4. ^1^Tunisia.

## 4. Discussion

The objective of the present study was to adapt some items of the CATCH scale to generate the CATCH-TSG (CATCH applied to Traditional Games) to fit the physical activity field. The CATCH scale (Rosenbaum et al., 1986a) has enabled and now enables researchers to measure children’s attitudes towards disability. The present study provides evidence of the validity of the CATCH-TSG scale, a CATCH adaptation to a physical activity environment for young and adult people in Arab, English, French, Italian, Polish, Portuguese, Russian and Spanish. Also, we created a short version facing both age groups, and all the languages. And the versions do not have the best-fit indices nor reliability coefficients, in each particular language and age version. But our adapted and reduced scale combines the best fit indices and reliability coefficients, using the same items and configuration of factors.

The three-factor solution initially provided by Rosenbaum et al. (1986a,b) for the CATCH was not replicated in the CATCH-TSG. This is not new, as many past studies found different solutions validating the original CATCH (Bossaert and Petry, 2013). On the other hand, it has also been proposed to introduce different channels, such as a video or a vignette of a peer with disabilities, to encourage change in attitudes (Beattie et al., 1997; Bossaert et al., 2011; Link et al., 2004; Manetti et al., 2001). However, we approximated the theoretical model proposed by Triandis (1971) because factors 1 and 2 can be interpreted as attitudes with more emotional contents, while the third one is a more cognitive factor. Finally, the behavioural component can be seen at factor 4. So, though without a perfect fit with the original solution, we agree with past studies (Armstrong et al., 2016; Vignes et al., 2008), which suggest that the CATCH scale is a complete instrument to assess the cognitive, affective and behavioural component of individuals towards disability, with good enough psychometric properties.

The present study is relevant because we adapted the CATCH into a physical activity context, and we tested whether a shortened version was possible. In fact, we found that the 19 items of the CATCH-TSG version were at least as valid as the full version. In general, reliabilities were good enough, though some languages, especially Arabian and Russian, demonstrated low reliabilities. However, according to Ziegler et al. (2014) short scales are good when we need to include them in comprehensive studies, and they have not demonstrated to be worse than the complete versions.

### 4.1. Limitations

It is necessary to consider several limitations when interpreting the results of this work. First, although the study was represented by a diverse set of countries and populations, the sample was within 200 subjects in some countries, thus restricting the variety of stigma responses. The sample had to be reduced because some answers’ quality was not the same since invented or false response patterns were observed. In addition, the effects of sample collection were affected in some countries due to the COVID-19 situation adding variability in samples.

Second, cultural differences mark different styles of attitude towards disability stigma. The geographical distribution of individuals also influences the information and quality of mental health services and the awareness and dissemination of stigma amongst individuals in society. On the other hand, in the case of the Arabic language, which obtained the worst reliability scores in the factors of the CATCH-TSG, it must be taken into account that it is a language with many localisms. The version of the test, in which a standard language was used may have interfered with the interpretation and understanding of the items.

Thirdly, the writing of the items can stimulate acquiescence (the tendency of responding to agree with the questions of the test without prior reasoning). However, this is an intrinsic problem with the original measure (Rosenbaum et al., 1986a). One method used to minimise this effect is the reverse coding items written negatively, so that high scores represent a more positive attitude.

### 4.2. Conclusion

We should increase the sample size and revise some translations in future studies. For this purpose, the survey collection system employed in this study exhibits robustness, leverages state-of-the-art technologies, and effectively fulfils the investigation’s requirements. Therefore, it is well-suited for utilization in future iterations of similar research endeavours. In addition, using a longitudinal design with repeated measures to develop future evidence-based clinical strategies is advisable. However, the present study must be taken as the first approximation to the validity and reliability of the CATCH test adapted to physical activity contexts. Considering this fact, we believe that the CATCH-TSG is valid and reliable enough to continue researching it.

## Data availability statement

The datasets presented in this article are not readily available because we do not yet have the authorisation of all the countries involved. Requests to access the datasets should be directed to JR-T, josep.riustorrento@udl.cat.

## Ethics statement

The studies involving humans were approved by the Ethics Committee for clinical research of the Catalan Sports Council; number 09/CEICGC/2020. The studies were conducted in accordance with the local legislation and institutional requirements. Written informed consent for participation in this study was provided by the participants' legal guardians/next of kin. Written informed consent was obtained from the individual(s) for the publication of any potentially identifiable images or data included in this article.

## Members of the Opportunity Team

Spanish Opportunity Team: Camacho R, Duran C, Esperanza M, Fernández-Amat C, Gracia I, Lagardera F, Mallén C, Muñoz-Arroyave V, Ormo E, Pla P, Prat Q, Rodriguez-Arregi R, Ruiz P, Sáez de Ocáriz U, Soria B; Portuguese Opportunity Team: Jaqueira AR, Coelho de Araújo P, Romao A, Rodrigues MDM; Tunisian Opportunity Team: Ben Chaâbanne Z, Bouzid E; Italian Opportunity Team: Avigo PG, Berti F, Carcereri L, Berti F, Torresani G; Croatian Opportunity Team: Đerić Nikolić T, Pacenti M, Perković T; Polish Opportunity Team: Prabucki B, Waluch K; Russian Opportunity Team: Kuznetsova Z.

## Author contributions

JM-L: Conceptualization, Investigation, Methodology, Software, Validation, Writing – original draft, Writing – review & editing. LM-R: Data curation, Funding acquisition, Methodology, Resources, Software, Visualization, Writing – review & editing. JM-H: Conceptualization, Investigation, Methodology, Software, Validation, Writing – original draft, Writing – review & editing. JR-T: Data curation, Funding acquisition, Methodology, Resources, Software, Visualization, Writing – review & editing. VE-P: Investigation, Supervision, Writing – review & editing, Visualization. JB: Investigation, Validation, Visualization, Writing – review & editing. PL-B: Conceptualization, Investigation, Project administration, Supervision, Writing – review & editing. The Opportunity Team: Translation into country Language, Data Collection, Writing – review & editing.

## Funding

This work was supported by the Ministerio de Ciencia e Innovación under contract PID2020-113614RB-C22 funded by MCIN/AEI/10.13039/501100011033, by the Agència de Gestió d'Ajuts Universitaris i de Recerca de la Generalitat de Catalunya (2021SGR1432) and by the Center for Biomedical Research Network on Mental Health (CIBERSAM), Instituto de Salud Carlos III. The study was also co-funded by the Erasmus+. Project of the European Union: Opportunity. Fostering Social Inclusion & Gender Equality through Traditional Games and Sports, code:622100-EPP-1-2020-1-ES-SPO-SCP.

## Conflict of interest

The authors declare that the research was conducted in the absence of any commercial or financial relationships that could be construed as a potential conflict of interest.

## Publisher’s note

All claims expressed in this article are solely those of the authors and do not necessarily represent those of their affiliated organizations, or those of the publisher, the editors and the reviewers. Any product that may be evaluated in this article, or claim that may be made by its manufacturer, is not guaranteed or endorsed by the publisher.

## Footnotes

^1^ http://opportunityproject.eu/

^2^ https://angular.io/

^3^ https://nodejs.org/en

^4^ https://www.mongodb.com/

^5^ https://www.docker.com/
