# Supplementary material for: Chedoke-McMaster attitudes towards children with handicaps scale for traditional sporting games (CATCH-TSG): initial validation in 7 different languages in adult and young populations
Source: Front Psychol. 2023 Sep 25;14:1254580. doi: 10.3389/fpsyg.2023.1254580 (PMC10561691; doi:10.3389/fpsyg.2023.1254580)
Supplement: Supplementary file 1 [file Data_Sheet_1.docx]

YOUTH version (under 18 years old)

CATCH-TSG Chedoke-McMaster Attitudes Towards Children with Handicaps (CATCH) Scale when participating in Traditional Games and Sports.

Validation reduced version based on Rosenbaum P, Armstrong R, King S. (1986). Children's attitudes toward disabled peers: a self-report measure. J Pediatric Psychol; 11,517–30.

Below are various situations in which you are asked to express your degree of agreement. There are 5 response options: 1. Strongly disagree; 2. Disagree; 3. Neither agree nor disagree; 4. Agreed; 5. Strongly agree.

Please select the option that best represents your degree of agreement. Answer all the sentences, even if you have any questions. There are not correct or incorrect answers.

| Factor | Items | Strongly disagree | Disagree | Neither agree nor disagree | Agreed | Strongly agree |
| --- | --- | --- | --- | --- | --- | --- |
| 3 | 1. Children with disability can practice traditional sports and games by themselves. | 1 | 2 | 3 | 4 | 5 |
| 2 | 1. I wouldn’t know what to say to a child with disability. | 1 | 2 | 3 | 4 | 5 |
| 3 | 1. Children with disability like to play. | 1 | 2 | 3 | 4 | 5 |
| 4 | 1. I feel sorry for children with disability. | 1 | 2 | 3 | 4 | 5 |
| 4 | 1. Children with disability require a lot of attention from adults when practicing traditional sports or games. | 1 | 2 | 3 | 4 | 5 |
| 2 | 1. I would be afraid of a child with disability. | 1 | 2 | 3 | 4 | 5 |
| 1 | 1. I would like having a child with disability on my traditional sport or game team. | 1 | 2 | 3 | 4 | 5 |
| 4 | 1. Children with disability feel sorry for themselves. | 1 | 2 | 3 | 4 | 5 |
| 1 | 1. I would be happy to have a child with disability as a special friend. | 1 | 2 | 3 | 4 | 5 |
| 2 | 1. I would try to stay away from a child with disability practicing traditional sports or games. | 1 | 2 | 3 | 4 | 5 |
| 3 | 1. Children with disability are as happy as I am practicing traditional sports or games. | 1 | 2 | 3 | 4 | 5 |
| 1 | 1. I would be pleased if a child with disability invited me to his house. | 1 | 2 | 3 | 4 | 5 |
| 3 | 1. Children with disability don’t have much fun participating in a traditional sport or game. | 1 | 2 | 3 | 4 | 5 |
| 1 | 1. I would invite a child with disability to sleep over at my house. | 1 | 2 | 3 | 4 | 5 |
| 2 | 1. Being near a child with disability when doing traditional sports or games scares me. | 1 | 2 | 3 | 4 | 5 |
| 3 | 1. Children with disability are interested in lots of things. | 1 | 2 | 3 | 4 | 5 |
| 2 | 1. I would be embarrassed if a child with disability invited me to their birthday party. | 1 | 2 | 3 | 4 | 5 |
| 3 | 1. Children with disability can make new friends. | 1 | 2 | 3 | 4 | 5 |
| 4 | 1. Children with disability need lots of help to practice traditional sports or games. | 1 | 2 | 3 | 4 | 5 |

ADULT version (From 18 years old)

CATCH-TSG Chedoke-McMaster Attitudes Towards Children with Handicaps (CATCH) Scale when participating in Traditional Games and Sports.

Validation reduced version based on Rosenbaum P, Armstrong R, King S. (1986). Children's attitudes toward disabled peers: a self-report measure. J Pediatric Psychol; 11,517–30.

Below are various situations in which you are asked to express your degree of agreement. There are 5 response options: 1. Strongly disagree; 2. Disagree; 3. Neither agree nor disagree; 4. Agreed; 5. Strongly agree.

Please select the option that best represents your degree of agreement. Answer all the sentences, even if you have any questions. There are not correct or incorrect answers.

| Factor | Items | Strongly disagree | Disagree | Neither agree nor disagree | Agreed | Strongly agree |
| --- | --- | --- | --- | --- | --- | --- |
| 3 | 1. People with disability can practice traditional sports and games by themselves. | 1 | 2 | 3 | 4 | 5 |
| 2 | 1. I wouldn’t know what to say to a person with disability. | 1 | 2 | 3 | 4 | 5 |
| 3 | 1. People with disability like to play. | 1 | 2 | 3 | 4 | 5 |
| 4 | 1. I feel sorry for people with disability. | 1 | 2 | 3 | 4 | 5 |
| 4 | 1. People with disability require a lot of attention from adults when practicing traditional sports or games. | 1 | 2 | 3 | 4 | 5 |
| 2 | 1. I would be afraid of a person with disability. | 1 | 2 | 3 | 4 | 5 |
| 1 | 1. I would like to have a person with disability on my traditional sport or game team. | 1 | 2 | 3 | 4 | 5 |
| 4 | 1. People with disability feel sorry for themselves. | 1 | 2 | 3 | 4 | 5 |
| 1 | 1. I would be happy to have a person with disability as a trusted friend. | 1 | 2 | 3 | 4 | 5 |
| 2 | 1. I would try to stay away from a person with disability practicing traditional sports or games. | 1 | 2 | 3 | 4 | 5 |
| 3 | 1. People with disability are as happy as I am practicing traditional sports and games. | 1 | 2 | 3 | 4 | 5 |
| 1 | 1. I would be pleased if a person with disability invited me to his house. | 1 | 2 | 3 | 4 | 5 |
| 3 | 1. People with disability don’t have much fun participating in a traditional sport or game. | 1 | 2 | 3 | 4 | 5 |
| 1 | 1. I would invite a person with disability to go out to dinner. | 1 | 2 | 3 | 4 | 5 |
| 2 | 1. Being near a person with disability when doing traditional sports and games scares me. | 1 | 2 | 3 | 4 | 5 |
| 3 | 1. People with disability are interested in lots of things. | 1 | 2 | 3 | 4 | 5 |
| 2 | 1. I would be embarrassed if a person with disability invited me to go out for a drink. | 1 | 2 | 3 | 4 | 5 |
| 3 | 1. People with disability can make new friends. | 1 | 2 | 3 | 4 | 5 |
| 4 | 1. People with disability need lots of help to practice traditional sports or games. | 1 | 2 | 3 | 4 | 5 |
